# Rethinking Depression—Beyond Neurotransmitters: An Integrated Psychoneuroendocrineimmunology Framework for Depression’s Pathophysiology and Tailored Treatment

**DOI:** 10.3390/ijms26062759

**Published:** 2025-03-19

**Authors:** Anna Giulia Bottaccioli, Mauro Bologna, Francesco Bottaccioli

**Affiliations:** 1 Department of Oncohematology, Clinical Psychology Graduated Course, University of Milan, I-20122 Milan, Italy; 2 Società Italiana di Psiconeuroendocrinoimmunologia, I-00195 Rome, Italy; mauro.bologna@univaq.it (M.B.); francesco.bottaccioli@gmail.com (F.B.); 3 Department of Medicine, Health, Life and Environment, University of L’Aquila, I-67100 L’Aquila, Italy; 4 Post-Graduated Course of Psychoneuroendocrineimmunology, Humanitas University Consortium Rome, I-00193 Rome, Italy

**Keywords:** depression, immune system, inflammation, integrated care, neurotransmitters, nutrition, psychiatry, psychoneuroendocrineimmunology, resistant depression, stress

## Abstract

It is known that the effectiveness of drug treatment for depression, ammine deficit based, is largely unsatisfactory. In this review, we examine the proposal of a precision therapy has emerged and has received a strong push by the identification of the role of inflammation in depression. However, precision psychiatry risks being caught in the reductionist trap of searching for the molecular switch that resets the whole system and switches off the disease. This is an illusion since the human being is complex and depression is a systemic and variable disorder. In this study, we show the inadequacy of the reductionist paradigm, and, at the same time, illustrate the superiority of the systemic paradigm centered on psychoneuroendocrineimmunology (PNEI). According to the PNEI paradigm, depression is a disease of the whole human being, caused by different sources working together: psychological, biological, and behavioral. This means knowing the biological and psychological history of the subject, identifying relational and biological crisis factors, and building personalized treatments targeting those factors with the tools of medicine and psychology, which are not reducible to the combination of drugs and psychotherapy. Our proposal presents a paradigm shift that is both theoretical and practical, which enables clinicians to assess patients experiencing depression in a unified way and treat them in an integrated manner.

## 1. Introduction

Depression is a major health problem worldwide, especially in countries with higher industrialization. All age groups are affected. In the USA, 18.1% of adolescents (12–17 years old) in 2023 had a major depressive episode (MDE, 4.5 million), 74.7% of which (3.4 million) had severe impairment. A total of 8.5% (or 21.9 million people) of adults over the age of 18 had an MDE: the percentage was highest among young adults aged 18 to 25. A total of 15.3 million people in 2023 had a MDE with severe impairment in the past year. Overall, it can therefore be said that 26.4 million people aged 12 and over in 2023 recorded a major depressive episode, which in most cases produced severe impairment. Over 60% are women.

A significant proportion of depressed adolescents and adults systematically use substances, or substance use disorder (SUD), and approximately 13 million adults in 2023 had serious thoughts of suicide, made a suicide plan, or attempted suicide in the past year [1].

In Europe, the prevalence of depression is 6.54% but post-COVID-19 (2020), there are considerable differences between countries: Greece 1.85%, Italy 4.29%, Germany 8.22, United Kingdom 9.26%, and Sweden 10.72% [2].

Together with young people, the elderly are at particular risk. Globally, the prevalence of depression in older adults is over 1/3 (35.1%) [3].

Depression is a chronic disease with significant effects on biology and human health.

Major depression disease (MDD) is associated with a relative risk of suicide 7.6-fold greater than non-depressed [4] and with an increase in all-cause mortality, including cardiovascular disease, and ischemic heart disease, among adults [5]. The economic burden of depression in the US was calculated in 2019 as the amount of USD 16,854 per adult with MDD with a total cost to society that exceeds 300 billion dollars due to numerous factors including healthcare costs and decreased productivity at work [6]. Despite decades of pharmacological research and a massive and increasing prescription of antidepressants, less than half of the treated subjects (48%) showed improvement (symptom remission) after 6 months of treatment, a percentage that drops to 34% if functional remission is assessed [7]. More recently, a Minnesota investigation determined that only 10.8% of patients experienced a functional remission after 6 months, a percentage that drops to 9.8 after 12 months [8].

Meta-analyses show that, at 12-month follow-up, psychotherapy is superior to pharmacotherapy and that combined therapy of antidepressant drugs and psychotherapy is superior to pharmacotherapy alone, but not superior to psychotherapy alone [9].

The traditional explanation of the pathogenesis of depression, centered on the imbalance of brain neurotransmitters (serotonin in the first place), is now judged unsatisfactory and still widely debated in the scientific community [10,11].

For those reasons, it is urgent to outline an integrated pathophysiological framework and tailored treatment based on the scientific vision of the human being as a whole, in which psychology and biology influence each other [12].

In this study, we will show the inadequacy of the reductionist paradigm, which has dominated research and clinical practice for half a century, and, at the same time, illustrate the superiority of the systemic paradigm centered on psychoneuroendocrineimmunology, which enables clinicians to assess patients experiencing depression in a unified way and treat them in an integrated manner. This means knowing the biological and psychological history of the subject, identifying relational and biological crisis factors, and building personalized treatments targeting those factors with the tools of medicine and psychology.

Currently, the union of medicine and psychology is made by the combination of medication and psychotherapy. This is a primitive form of integrated therapy that is not based on the recognition of the subject’s different crisis factors and their relative weight, which include eating, motor, and environmental behaviors, and, above all, it gives little value to interventions that mainstream medicine does not consider therapeutic, but at most complementary. In fact, for some patients, a change in diet may be of greater therapeutic value than the addition of a new antidepressant. Thus, action on the patient’s biology cannot be limited to drugs, the level of efficacy of which is modest, but can include physical activity, personalized nutraceuticals based on biomarkers, and a specialized non-pharmacological medical intervention such as acupuncture, which one of the authors (A.G.B.) also uses routinely in psychiatric disorders as we shall see in the clinical case we present.

Our proposal presents a paradigm shift that is both theoretical and practical, based on the union of medicine and psychology [12].

## 2. Rise and Decline of the Standard Vision of Depression, Based on an Imbalance in Neurotransmitters

The theory that has dominated for the past four decades considers depression to be the result of a chemical imbalance, particularly of neurotransmitters such as serotonin and norepinephrine. It is the so-called monoaminergic theory of depression that, with more difficulty, has also been attempted to be applied to psychoses (dopamine receptor alterations). In the early eighties, biological psychiatry became a scientific ideal in the United States that quickly conquered psychiatrists, doctors, and the public.

Anne Harrington. historian of science, recalls that 1984 was a turning point [13]. The influential psychiatrist Nancy Andreasen published a book that is the manifesto of biological psychiatry, The Broken Brain: The biological Revolution in Psychiatry, where one can read: “psychiatry is undergoing a revolutionary change, of realignment with the biological traditions of medicine” [13] (p. XII). (See Supplementary File on the history of the standard vision of depression).

Biological psychiatry is based on the following concepts: (1) mental disorders are disorders caused by a malfunction of the brain; (2) the causes are genetic; (3) the only therapy is pharmacological; psychotherapy can only be supportive [14,15].

With the beginning of the new century, evidence has accumulated that does not confirm the three pillars of the standard view.

The efficacy of antidepressant drugs has been questioned since the first meta-analyses of the late 1990s [16]. Subsequent research, of which we wrote about in the Introduction, confirmed that the effectiveness of antidepressants is modest. A systematic review and network meta-analysis, carried out by a high-level international team and funded by the National Institute for Health Research Oxford and the Japan Society for the Promotion of Science, which is presented as the main evidence of the efficacy of antidepressants, warns: “We found that all antidepressants included in the meta-analysis were more efficacious than placebo in adults with major depressive disorder and the summary effect sizes were mostly modest” [17] (p. 1362, italics our)—a modest effect that is, however, difficult to detect in adolescents, in whom “fluoxetine is probably the only antidepressant that might reduce depressive symptoms” (ibid.).

The low effectiveness of antidepressants that enhance the intrasynaptic availability of serotonin and other neurotransmitters cannot therefore prove that the cause of depression is a deficiency of these molecules.

In addition, a systematic umbrella review, which examined the major areas of research regarding the hypothesis that serotonin is deficient during depression, found no evidence that brain deficiency or low activity of the neurotransmitter is at the root of the psychiatric disorder [10]. This research has also reviewed work related to the genetics of depression, which is the third pillar of the standard view of depression, although the gene alterations responsible for the disorder have never been found. However, at the beginning of the present century, the international community of psychopharmacological research has welcomed, with great enthusiasm and hope, the studies presented by A. Caspi [18], which no longer emphasize genes, but the polymorphism of an area (that of the promoter) of the gene that controls the synthesis of the serotonin transporter (5-HTTLPR). From the first works, it appeared that the owners of the short variant of the promoter, especially if in homozygosity (S-S), were predisposed to depression, from which the owners of the long variant (L-L) were protected. In fact, subsequent research, including that of Moncrieff cited above, has not confirmed this relationship. After all, Caspi himself warned against determinism “which is dangerous. Instead, it is a question of embracing a more realistic and refined understanding of the causes of behavior, in which the effects of some genes depend on lifestyle choices”, including stress [19].

In conclusion, we can state that the standard model of depression and mental disorders in general lacks convincing explanations of the pathophysiology and lacks effective drugs, which, discovered occasionally (serendipity), have been the same since the middle of the last century. This explains the very low efficacy and efficiency rate of current pharmacological treatments [20].

## 3. A New Pathophysiological Framework: The Central Core

As we have seen above, the theory that depression derives from a deficiency of cerebral serotonin of genetic origin has no evidence; however, the supporters of the chemical imbalance extend the chemical factors of imbalance to serotonin, norepinephrine, dopamine, glutamate, hypothalamic–pituitary–adrenal (HPA) axis, GABA, glucocorticoids, inflammatory cytokines and brain-derived neurotrophic factor (BDNF) [21]. These other biochemical and cellular modifications (which also affect microglia and astrocytes) are plausible; we also add that during depression, the activity of fundamental brain circuits is altered, as is documented by studies with brain images that have shown an expansion of the frontostriatal circuit of salience in depressed people [22]. But what does this mean? It is quite evident that depression produces an alteration in cerebral biology, cells, molecules, and circuits, as well as an alteration in other biological systems, including metabolic, immune and endocrine system, adipose, muscular and bone tissues. These concepts are trivial in the current scientific era. Just as it is quite evident that a depressed person is affected by anhedonia, asthenia, brooding over negative thoughts, social withdrawal, negative expectations of oneself and the world, sleep and eating disorders. Is it possible to separate these psychological conditions and behavior from the biological alterations mentioned above? No, it is not possible to separate biology and psychology, nor is it possible to establish a one-way supremacy of biology over psychology. In fact, it is scientifically documented that trauma, stress, minority stress, adversity since early stages of life can alter biology, through epigenetic signatures, and that these biological alterations, in turn, can influence the psychic state [12,23,24,25,26,27,28,29].

It seems clear to us that depression is a disease of the human being in its entirety. The sources of the disorder, as we will see in the next paragraphs, can be biological, psychological, relational, and environmental; and once activated, they synergize with each other. An inflammatory state from obesity and a sedentary lifestyle, through the signaling to the brain of cytokines and molecules of the microbiota and adipose tissue, can induce an alteration in mood and cognition, which, in turn, increases inflammation. Similarly, a condition of psychological suffering, such as stress, loneliness, poverty and social isolation, can alter the immune system in an inflammatory sense with the aforementioned consequences on mood.

Not embracing this paradigm, that found the research in psychoneuroendocrineimmunology, involves two errors: one philosophical and the other scientific. The philosophical one, already described by George Engel [30], consists of reducing a complex phenomenon, such as depression, to a simple determinant (a molecule, a gene, an epigenetic signature) and to a single level (the biological one). The scientific one, which derives from the first, consists of restricting the field of research to the obsessive and frustrating search for the right molecule of the switch that will allow the entire network to be reset. The reductionist model does not allow the researcher to understand the reciprocal interrelationship, the synergy, between psychological, social and biological factors and does not allow the clinician to address the disorder with diversified tools that act on psychology and biology. In the era of generative artificial intelligence (AI) capable of producing knowledge by integrating information from multiple levels and extracting generalizations of higher quality, adopting a single-level reductionist model, the biological one, condemns clinical research to remain in the stagnation of recent decades, in which progress has been lower than expected and not corresponding to the economic investments made. Integrating knowledge is the new frontier that AI proposes to us, as documented by research that has compared AI models to expert neuroscientists on the ability to predict results [31]. From this experimentation, it emerges that the superiority of large language models (LLM) over human experts came from integrating information, not from the storage of individual data. This could enhance multidisciplinary communication [32] and provide a unitary, higher-level view of human functioning and its disorders.

Combining the patient’s psychological and biomedical data into a framework improves the knowledge of the disorder sources, their interaction and above all allows the setting up of an integrated treatment that combines medical and psychological therapeutic resources. In paragraphs 3 and 4, we will see the assessment and the integrated treatment details. In particular, in the clinical case of severe major depression that we will illustrate later, we document how, in the face of the failure of standard treatment, based on antidepressants and psychotherapy, it was possible to bring the patient out of depression by combining anti-inflammatory nutrition, personalized nutraceuticals, meditation training, physical activity and acupuncture. This involves a new vision of integrated treatment, which cannot be the addition of psychotherapy to antidepressants. This therapy, also known as combined therapy or even integrated therapy, is still a narrow vision, which does not leave the reductionist model and which for this reason does not give superior results to psychotherapy alone. To set up a true integrated therapy, we need to know the major sources of depression, biological, psychological, and social.

### 3.1. The Depression Sources

In the third decade of the twenty-first century, five planetary phenomena have come to maturity with a powerful charge of disturbance of human life and of the ecosystems, which, influencing and mutually supporting each other, produce systemic effects: (1) climate change; (2) massive transmigrations of human beings and social crisis in rich countries; (3) disseminated world war; (4) growth of inequalities between nations and within nations; (5) epochal technological leap (artificial intelligence). The systemic effects of these phenomena affect all aspects of life, individual, economic, social, and political. On an individual psychic level, instability, uncertainty, anxiety, disorientation, and difficulty in imagining a future, are the fundamental aspects. Recent research shows that technological innovation, which has allowed the planetary development of social media in the last 15 years, has a significant effect on the psyche in particular on the youngest, defined as the “anxious generation” [33,34]—effects that will presumably be enhanced by the rapid development of artificial intelligence applied to the construction of virtual environments and extended reality, which will increase the pressure on individuals involved in cognitive, relational and occupational changes, even of considerable magnitude [35,36]. People, therefore, live and will increasingly live in a condition of stress in the near future.

#### 3.1.1. Stress: General Concepts, and Relevance to Depression

In the last hundred years, research has framed the adaptation of the human being to the challenges of life and possible suffering within the concept of stress [37,38,39,40,41,42,43]. Hans Selye [37,38,39], for the first time, introduced some fundamental concepts: (1) stress can be protective or harmful; (2) stressors induce an adaptation to environmental stimuli, which can be adequate or inadequate, stimulating, in the latter case, diseases of adaptation; (3) if there is maladaptation, the organism is marked by the accumulation of products of biological activity; (4) stress management is purely individual.

These concepts were found and developed in subsequent research, although ironically the main innovators, such as Paul Sterling [40] and Bruce McEwen [41], will criticize Selye by attributing to him a static, homeostatic vision. Sterling, criticizing the homeostatic vision, which in our opinion was Walter Cannon’s and not Hans Selye’s ([39], in 1976, thus well before the emergence of the concept of allostasis, proposed the concept of “heterostasis” (from the Greek *heteros* or other) which documents his dynamic view of homeostasis. According to Selye, the difference between homeostasis and heterostasis is that the latter requires to “reset the thermostat” and therefore a change that, for Selye, was being prompted by an external intervention, for example by drugs. [39] (p. 31)), proposes the concept of allostasis defined as “achieving stability trough change”. The next scientific passage is by Bruce McEwen and other researchers: since the chronic stress of allostatic mechanisms can lead to disease, he introduced the concept of “allostatic load or overload”, that current scientific research is actively engaged in measuring to use it as a predictive model of disease [42].

According to Selye, environmental stressors in the human being are eminently emotional, but not only, since they depend on the individual type of life management, sleep, nutrition, pollution and the ability to cultivate social relationships as well as on the ability to cultivate techniques that improve physical and mental fitness, such as physical activity, Yoga and Meditation [38] (p. 453). Mastering life tends to reduce the harmful effects of stress, which is inevitable as it is the very essence of life. However, good stress management can also be an opportunity for “growth experiences”, to improve vital balance. This type of stress is called eustress (good stress). If, on the other hand, stress overwhelms the subject’s internal and external resources that prevent the control and management of stressful events, we are faced with distress. Bruce McEwen introduced a third condition called “toxic stress” by linking it to stressful events in the early stages of life. “Toxic stress refers to an individual who has limited support and who may also have brain architecture that reflects effects of adverse early life events that have impaired the development of good impulse control and judgment and adequate self-esteem” [43] (p. 2).

We will see later the role of early life adversity on the brain, mental states, and immunity: here it is useful to briefly recall the organization of the stress system, and the mediators involved.

**Figure 1:**
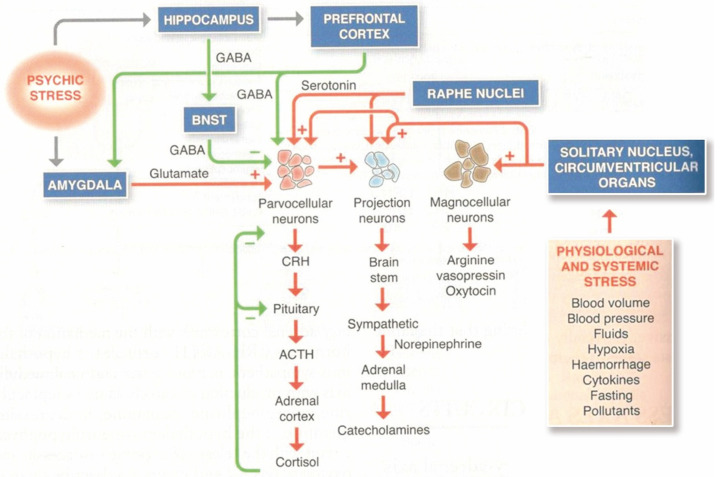
The stress system. The paraventricular hypothalamic nucleus is the cerebral structure that activates stress response. It is divided into three sectors: magnocellular, which releases oxytocin and arginine vasopressin; parvocellular, which releases CRH; and the neurons projected towards the brain stem, where the nuclei activating the neurovegetative system are located, the locus coeruleus and the rostral ventromedial medulla, in which the control nuclei of the sympathetic nervous system are located. The stress system can be activated both psychological and endogenous and environmental stressors. This peculiarity shows how biological (cytokines, other biomolecules, and internal states) and environmental (pollutants) stressors can affect the brain and mind and thus be part of the pathogenesis of mental illness [44] (p. 136). With Edra license.

Researchers usually adopt the term “stress axis” to describe the stress system. Stress axis is a restrictive term since stress activates a hypothalamic platform, composed of various nuclei, involving numerous mediators that are networked together, with far-reaching central and peripheral effects. In addition, it is good to keep in mind that the stress system can be activated by psychic, social, endogenous, and external environmental stressors. Figure 1 illustrates the main dynamics.

The network of mediators is a “nonlinear regulatory network” [45]; thus, the same mediator can have different effects depending on the context of the network and different target tissues. For example, norepinephrine has peripheral anti-inflammatory effects, while centrally it can activate neuroinflammation, via microglial cells [46].

#### 3.1.2. Stress and Brain: Mechanisms Based on Brain Immunity

In the late 1960s, the discovery of the brain receptor for glucocorticoids by Bruce McEwen [47] started a long phase of research to unravel the effects of stress in the brain. This research, which investigated the effects of hyperactivation of the neuroendocrine stress axis (HPA), had a milestone in documenting the pathogenic role of excess cortisol on important brain areas such as the medial prefrontal cortex (mPFC), the orbitofrontal cortex, the hippocampus and the amygdala. From this research, then consolidated over time [23,48], it emerged that the brain, under chronic stress, undergoes functional and structural alterations, with atrophy of the mPFC, the hippocampus and a hypertrophy of the orbitofrontal, while the amygdala seems to undergo a contrasting alteration: hypertrophy of the basolateral nucleus and hypotrophy of the central nucleus. The overall effects of these alterations are increased emotional hyperactivity (orbitofrontal and basolateral hyperactivity of the amygdala not moderated by the central amygdala equipped with a robust network of GABAergic neurons), memory lability (hippocampal hypotrophy), and cognitive and environmental self-regulation difficulties (mPFC and hippocampal hypotrophy and, consequently, of salience networks, which is based on these areas).

With the most recent findings on the role of immunity in the brain, the picture is complete. We know that not only can the brain be a target of the peripheral immune system, but also that the organ itself is home to a broad and diffuse immune system, present in the meningeal membranes, choroid plexus, cerebrospinal fluid and even directly in the brain parenchyma [49,50]. Microglia is a more relevant group of brain cells. From the earliest stages of embryonic brain formation, microglial cells migrate into the brain tissue and become resident cells capable of self-renewal; they account for 5% to 12% of all cells in the central nervous system and, unlike other nerve cells, have a mesodermal and not an ectodermal origin [51]. They are and therefore behave as macrophage-type myeloid lineage cells. These cells play fundamental roles, both in brain development by promoting the maturation of neurons and oligodendrocytes [52] and in the adult brain, by performing functions typical of macrophages such as phagocytosing various types of cellular debris and pathogens, but also regulating and maturing synapses through phagocytosis and the release of various factors, including cytokines, chemokines and extracellular vesicles [53,54]. Microglia, together with Mast cells, which are the other innate immunity cells residing in the brain, and also non-immune cells such as astrocytes, release cytokines and growth factors that perform physiological functions by promoting plasticity, learning and memory. However, the microglia–mast cell pair is a device that can be highly inflammatory if they become the target of inflammatory cytokines, which can originate from the periphery, or even of stress hormones.

In a chronic stress scenario, indeed, norepinephrine and cortisol overproduction can activate microglia and mast cell trough NLRP3 inflammasome, with release of pro-inflammatory cytokines, such as IL-1β and TNF-α, and Reactive Oxygen Species (ROS) [55], causing neuroinflammation and depression [56]. The combination of cortisol and inflammatory cytokines is most likely at the origin of the reduced hippocampal neurogenesis induced by hypoactivity of the dentate gyrus of the hippocampus under stress conditions [57]. Reduced hippocampal neurogenesis and hippocampal volume worsens the ability to cope with stressful events, reducing cognitive flexibility and favoring the acquisition of fear memory, with anxiety, and depression [58], and can be related to depression suicidal ideation.

Neuroendocrine and neuroimmune research together allow us to conclude that stress, understood as distress or toxic stress, through the systemic effects of cortisol and norepinephrine, is capable of altering brain activity, by means of various mechanisms, still to be fully discovered in their complexity, that seem to center on two fundamental pathways: alteration of the structure of brain areas and circuits, induction of neuroinflammation and oxidative stress. These neurobiological alterations support depression, anxiety and other psychiatric disorders which, in turn, by chronicizing stress and structuring proinflammatory lifestyles (see below), reinforce and deepen them.

#### 3.1.3. Early Life Adversities, Trauma, Loneliness and Depression

Research that began half a century ago shows that, during the early stages of life, environmental conditions can cause epigenetic changes that tend to persist for the rest of an individual’s life. Adverse environment can be biological, as in the case of exposure to undernourishment in the prenatal period that occurred in the Netherlands in 1944–1945 [59], or even affective, as in the case of mice reared by “caring” mothers (high-level LG-ABN licking and grooming and arched-back nursing) or by non-caring mothers (low-level LG-ABN). In the first case, the study of the subjects who had suffered starvation in the womb, documented that at the age of 60, they had alterations in the methylation of the gene coding for IGF-2 and the entire genome [60,61]. In the second case, mice reared by “negligent” mothers exhibited hypermethylation of the promoter gene for the hippocampal glucocorticoid receptor and thus under regulation of these receptors which are essential for proper functioning of the stress axis [62]. Relevant to this research is the fact that, when mice born to caring mothers (high-level LG-ABN) were moved to the cage of negligent mothers (low-level LG-ABN), epigenetic signaling was hypermethylation of the gene, demonstrating that it is maternal behavior that marks the animals’ brains. In the following decades, human studies have confirmed animal studies, documenting epigenetic alteration of the HPA system and depression [63].

**Figure 2:**
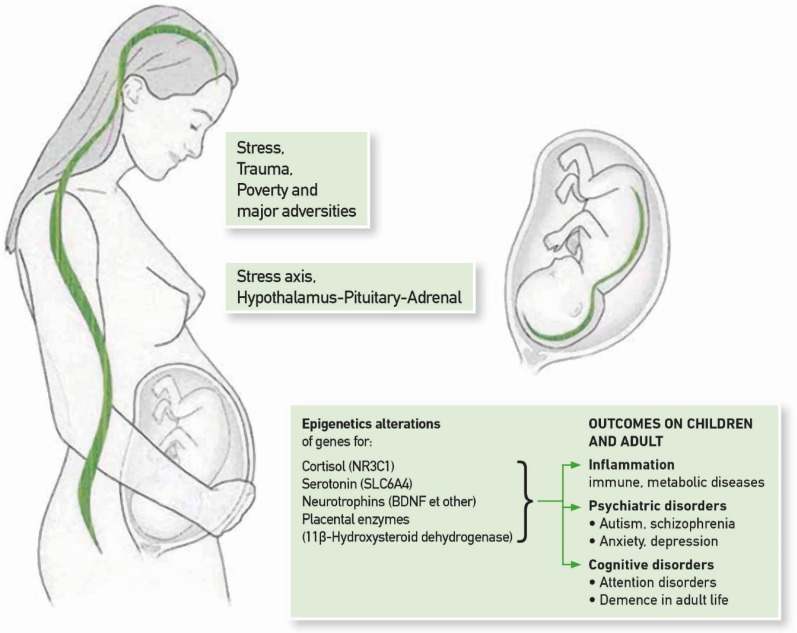
Stress in pregnancy: consequences for the fetus. Stress, trauma, poverty and other adversities in pregnancy can cause epigenetic alterations affecting key molecules for the health of all systems, but particularly the central nervous system of the developing fetus. NR3C1 is the gene coding for the receptor for cortisol; SLC6A4 is the gene coding for the serotonin transporter; BDNF is the gene coding for the eponymous molecule that is one of the main growth and plasticity factors in the brain in general and when developing in particular; the genes coding for 11-β hydroxysteroid (11-β-HSD-1,2) produce enzymes that moderate the passage of maternal cortisol across the placenta to the fetus: HSD-2 in particular transforms maternal cortisol into its inactive form. Hypermethylation of this gene reduces the enzyme’s activity and exposes the fetus to an excess of maternal cortisol, which can have detrimental effects on the fetus’ development.

Stress during pregnancy is a relevant line of research on the epigenetic modulation of fetal development. Maternal stress is associated with an internal inflammatory environment that epigenetically marks the stress axis and some key molecules in the fetus. Stressful conditions in pregnancy also due to poverty and low social status, as well as anxiety, depression and poor nutrition correlate with epigenetic alterations in the fetus and affect key molecules and systems: the neuroendocrine stress axis (with the NR3C1 gene coding for the cortisol receptor), the serotonin circuit (with alteration of the gene coding for the serotonin transporter, SLC6A4), the oxytocin circuit (OXTR) and the brain plasticity circuit (BDNF). Finally, stress in the pregnant woman alters the placental protective system of the fetus, based on an enzyme that controls the amount of maternal cortisol passing into the fetal environment (11β-hydroxysteroid dehydrogenase, 11β-HSD-2), which converts maternal cortisol into the less active cortisone. Epigenetic disruption of this enzyme system exposes the fetal brain to an excess of cortisol with possible extensive long-term destructive effects [64,65]. Figure 2 illustrates the mechanisms.

There is a body of research, which can be summarized in the category adverse childhood experience (ACE) or early life adversity (ELA), which encompasses different forms of abuse, physical, psychological, and sexual, which emphasizes how being exposed to adverse childhood experiences increases the likelihood of the onset of mental or physical illnesses in later life, up to old age [66,67]. In particular, about depression, there is growing evidence of a link between ACE and depression as adults, which is mediated by inflammation, recorded through increased IL-6 and TNF-α, but not by PCR [68]. Furthermore, a recent meta-analysis documented alterations in superior frontal, lingual gyrus, hippocampus, insula, putamen, superior temporal, inferior temporal gyrus, and anterior cerebellum in the brains of patients with major depression who had a history of ACE [69].

In this line of research, studies that have identified having experienced trauma, whether emotional, physical, sexual or physical abandonment, before the age of 18, as a major risk factor for adult depression should be considered. Trauma in childhood and adolescence is linked to a greater severity of depressive symptoms and resistance to treatment, although recent systematic investigations have shown that notwithstanding that symptomatology is higher in depressed patients with a history of trauma, offering evidence-based psychotherapy and pharmacology allows for improvement [70].

Depression and loneliness are intimately linked. Being isolated, with few social ties, or even feeling lonely, despite living in an adequate social and family context, is probably the most distressing and psychologically dangerous condition for human health. People who feel lonely are in a permanent state of alertness, afraid of others, of their judgements, of being rejected, feeling guilty or without perspectives. Research shows that loneliness and social exclusion, in the elderly, in males and females in their 40s, and in children, in addition to the psychological state described above, are associated with increased levels of inflammatory markers and a marked reactivity of the immune system to stressors both social and environmental (pathogenic microorganisms).

Steve Cole’s seminal research has shown that chronic activation of the stress system, caused by a condition of loneliness or social isolation, induces a ’conserved transcriptional response to adversity’ (CTRA) in immune cells. This is characterized by increased expression of pro-inflammatory genes (IL-1β, IL-6 and TNF-α) and decreased expression of anti-viral and antibody genes (e.g., IFNs) [71]. A meta-analysis that reviewed controlled studies on loneliness and social isolation found an association between loneliness and increased IL-6 concentration and between social isolation and fibrinogen [72].

The effects of loneliness, understood as the subjective perception of isolation, and social isolation, understood as the reduction in social contacts for various causes, on the inflammatory dysregulation of the immune system are at the root of numerous health problems: psychological, such as anxiety depression and suicidal ideation [73] and medical, such as atherosclerosis and cancer [74].

Loneliness has considerable effects on the brain. A systematic review of 41 loneliness studies (over 16,000 participants), in which different brain imaging technologies were used, showed altered structure and/or function in the medial and dorsolateral prefrontal cortex, anterior insula, amygdala, hippocampus, and posterior and superior temporal cortex [75]. The same systematic review documented a relationship between loneliness and an increased risk for the onset of dementia, as well as an increase in biological markers (beta-amyloid and tau protein) associated with Alzheimer’s disease (AD). An association between loneliness and AD has recently been further documented and it is recommended to assess it in any study on cognitive impairment in the elderly [76]. Furthermore, loneliness is correlated with various metabolic alterations, such as type 2 diabetes mellitus, dyslipidemia, metabolic syndrome, hypertension and other cardiovascular risk markers [77]—in sum, loneliness associated with inflammation and its consequences on psychological and medical disorders.

### 3.2. Inflammation, Nutrition, Microbiota, Physical Activity, and Pollution

In the previous paragraphs, we have repeatedly pointed out the relevant role of inflammation in psychic suffering. Immune dysregulation, which produces sterile inflammation, can be caused by distress, and toxic stress, as well as by autoimmune inflammatory diseases, intestinal dysbiosis, hypercaloric nutrition and junk food, obesity, sedentariness and indoor and outdoor pollution. It is therefore useful to keep in mind that inflammation can be caused by biological as well as psychological and behavioral factors.

#### 3.2.1. Inflammation and Depression

The vicious circle between stress, inflammation and depression has also been extensively analyzed in molecular detail over the past 25 years [78,79]. Some authors describe depression as a neuroimmune disorder, characterized by immune-linked neurotoxicity [80].

Although it is still unclear how many depressed people are affected by inflammatory phenomena, a significant proportion have clear signs of inflammation in their blood, with an increase in C-reactive protein (CRP) and inflammatory cytokines. A meta-analysis of mean differences and variability in 5166 patients and 5083 controls showed that levels of CRP, IL-3, IL-12, IL-18, sIL-2R and TNF were significantly higher in patients with depression [81]. In peripheral blood monocytes, the values of inflammatory cytokines in people with depression and controls were studied with surprising results. For example, the difference in the amount of IL-6 in the group with depression was 90-fold greater than in controls (978.1 pg/mL versus 11.1 pg/mL) [82]. Using 3 mg/L CRP as a threshold value (cut-off), approximately 40% of cases with depression had increased immune cell counts, inflammatory proteins and symptom severity scores, compared to the remaining 60% of cases without inflammation. However, a closer analysis of patients with depression could document that the proportion of depression cases associated with inflammation was higher and underestimated by the CRP cut-off. This research highlights the need for a more in-depth and refined study of the different forms of inflammatory depression, a frequent phenomenon during depressive disorder.

More recently, a survey on the immune profile of people at the first episode of major depression documented a significant activation of immune circuit Th1 compared to the control group, with an increase in inflammatory cytokines such as TNF and IL-6 and also several chemokines [83]. Finally, a survey using data from the National Health and Nutrition Examination Survey (NHANES), which included 2514 depressed and 26,487 non-depressed adults who were not hospitalized, found a direct and significant relationship between systemic inflammation, as measured by special indices, and depression [84].

We have already reported above on the role of the stress system in the production of neuroinflammation by microglia and other brain immune cells [85,86]. Indeed, a series of studies, using in vivo brain imaging in both animal models and humans, has documented, in the course of depression, an increase in key brain areas of a marker of inflammatory activation of microglia. The inflammatory marker detected was translocator protein (TSPO), the concentration of which, detected by positron-emission tomography (PET), was found to be increased in several brain areas of depressed subjects, including the prefrontal cortex, anterior cingulate, insula, amygdala and others [87,88], signaling widespread neuroinflammation.

However, inflammatory cytokines can reach the brain, via the blood circulation, vagus nerve and lymphatic system, from the peripheral immune system [89,90]. Robert Dantzer’s research, summarized in a seminal paper two decades ago [91,92], documented that peripheral infectious inflammation releases inflammatory cytokines that alter brain activity producing sickness behavior and depression. More recently, other research has highlighted the role of peripheral inflammation in depression in people with autoimmune diseases [93] and cancer [94]. Furthermore, a recent, crossover and prospective study (with 5 years of follow-up) documented that elevated blood levels of inflammatory cytokines (TNF, IL-17) are positively associated with the presence of depression in women. The same study was able to establish that elevated IL-17 levels are positively associated with the onset of depression in men at 5-year follow-up [95].

#### 3.2.2. Nutrition, Nutraceutical and Gut Microbiota

In recent decades, nutrition has become a topic of great interest in psychiatry [96] both because people in emotional distress and with psychiatric disorders usually have a disordered and poor diet, and because it is now clear that diet can influence mental states and psychiatric symptoms.

We have known for some years that excessive caloric intake causes inflammation of the hypothalamus [97,98], a brain area crucial for both energy balance and the endocrine system, including the stress control system [99]. One of the main highlighted mechanisms of hypothalamic inflammation, caused by a high-calorie, high-fat diet, is the increase in the intestinal hormone glucose-dependent insulinotropic polypeptide (GIP), which promotes hypothalamic inflammation and insulin resistance [100]. In addition to the central inflammatory action of GIP, one must consider the alteration and expansion of adipose tissue, which, by various mechanisms, functions as a center producing inflammatory cytokines reaching the brain [101].

Completing the picture is the role of the gut microbiota, which comprises vast ecosystems composed of an exorbitant number of microbes, including viruses, fungi and bacteria. They play several important roles in digestion, metabolism, synthesis of vitamins and short-chain fatty acids (SCFAs) and immune regulation. Numerous neuroactive substances are released from the microbiota and through the blood circulation and the fibers of the autonomic nervous system (the vagus nerve in particular) reach the brain. These substances are neurotransmitters (GABA, dopamine, noradrenaline, acetylcholine, serotonin), short-chain fatty acids (SCFAs), including acetate, butyrate, propionate and lactate, bile acids and tryptophan metabolites.

**Figure 3:**
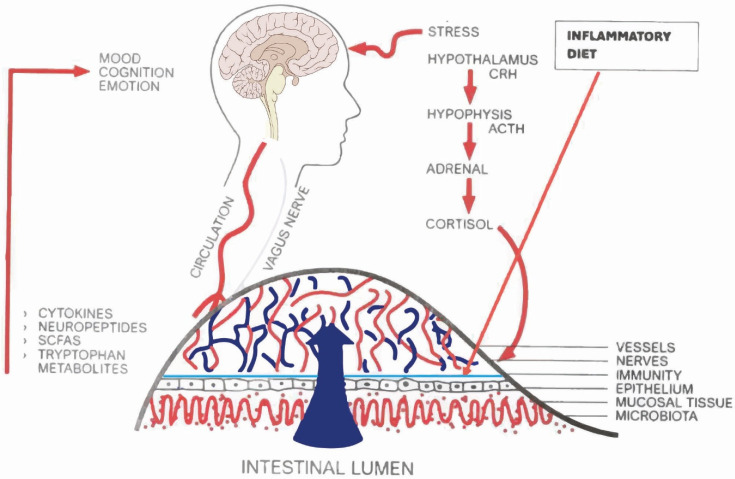
The relationship between gut, stress and mood. The figure shows that emotional stress, through the release of cortisol from the adrenal cortex, can alter the intestinal barrier, causing dysbiosis and intestinal inflammation, which, through the release of cytokines, fatty acids and other molecules, can in turn reach the brain, altering mood and cognition. Thus, chronic stress or inflammatory diet can simultaneously produce intestinal and mental disorders, including cognitive ones. CRH, corticotropin-releasing hormone; ACTH, adrenocorticotropic hormone; SCFAS, short chain fatty acids. From [12] (modified).

Furthermore, the intestinal mucosa hosts a very large immune system called gut-associated lymphoid tissue (GALT) that is in close contact with the microbiota and controls its potentially pathogenic strains. An excessive presence of the latter can cause an inflammatory response that can be exacerbated by a disruption of the intestinal barrier. Stress, with overproduction of cortisol, makes the gut walls more permeable (leaky gut) to microorganisms by increasing pro-inflammatory cytokines that in turn can send signals to the brain via the circulatory and autonomic nervous systems [102]. Figure 3 illustrates this fundamental mechanism that connects stress to the gut and from there to the psyche-brain system.

Alterations in the gut microbiota have been linked to mood disorders, anxiety and depression, schizophrenia, bipolar disorder, anorexia nervosa and other neuropsychiatric disorders such as Alzheimer’s disease, Parkinson’s disease, autism spectrum disorders and multiple sclerosis [103]. The gut microbiota of patients with major depression shows profound taxonomic changes associated with increased pro-inflammatory bacterial products, reduced synthesis of short-chain fatty acids (SFCAs), reduced integrity of the intestinal barrier and altered neurotransmitter production [104]. Intestinal dysbiosis, by increasing inflammation, can alter the tryptophan pathway with increased indoleamine 2,3-dioxygenase (IDO) activity that reduces the tryptophan metabolism to serotonin and increases the kinurenine pathway with overproduction of quinolinic acid (QA), which has inflammatory effects in the brain [105]. At the same time, inflammation is increased by the reduction in butyrate-producing bacterial strains. This SCFA has regulatory effects on the immune system and, at the same time, acts as a brain modulator via epigenetic mechanisms based on the activation of inhibitors of histone deacetylases (HDACis) [106].

The relevant role for the brain of diet-derived metabolites metabolized by the microbiota suggests that a low-grade inflammation diet may be important for mental health. The most studied low-grade inflammation diet in the world is the Mediterranean diet, which may positively influence mental health by modulating inflammation, oxidative stress, microbiota metabolites and other molecular signals from peripheral systems to the brain. In this regard, we have a relevant body of evidence.

A large study of approximately 2 million people documented that following a Mediterranean diet significantly reduced the risk of depression, lowering it by an average of one-third [107].

Another study of approximately 900 people with an average age of 73 years, who were free of depression at the start of the five-year study, showed that adherence to the Mediterranean diet is protective against the onset of depression and cognitive impairment. The consumption of fruit and vegetables was particularly beneficial in this study, along with moderate alcohol consumption (wine preferred) [108].

In children and adolescents, greater adherence to the Mediterranean diet also increases protection against mental suffering [109].

Overall, there is growing epidemiological evidence that a diet with higher consumption of fruit, vegetables, olive oil, oilseeds, fish and whole grains and lower consumption of meat, meat products, bakery products, trans fats, desserts and sugary drinks may reduce the risk of depression [110]. Finally, the results of a meta-analysis of randomized controlled trials that included the Mediterranean diet in the treatment of depression were recently published, involving more than 1500 patients. The authors concluded that the Mediterranean diet ’has substantial potential for alleviating depressive symptoms in people experiencing major or mild depression’ [111].

Finally, of great interest is the growing number of studies on vitamins, prebiotics, probiotics and food supplements used as part of an integrated depression therapy.

#### 3.2.3. Nutraceutical

##### Probiotics

Many microorganisms are called psychobiotics due to their activity on the psyche [112]. Randomized controlled studies have demonstrated the efficacy of various probiotic species in the treatment of depression, among them in particular *L. helveticus*, *L. rhamnosus*, *B. longum* and *B. breve* [113].

A review, which looked at controlled animal and human studies, concluded that the use of a probiotic mixture of Bifidobacterium strains has antidepressant effects, which are traced back to the anti-inflammatory, stress-axis-regulating and brain serotonin synthesis actions performed by these microorganisms [114]. An umbrella meta-analysis that looked at 10 meta-analyses including more than 8000 subjects reported robust effects of probiotics on depressive symptoms in adults, but only if the treatment involved the daily intake of more than 10 billion live microorganisms and lasted for more than 8 weeks [115].

It can be concluded that the use of probiotics, as part of a low-grade inflammation diet, may be useful in the integrated treatment of depression.

##### Omega-3

Meta-analyses have shown that, in people suffering from depression and other psychiatric disorders, there is a reduction in long-chain polyunsaturated fatty acids (lcPUFAs) in cell membrane [116]. Furthermore, eicosapentaenoic acid (EPA) has a documented anti-inflammatory activity that may be useful in controlling depressive symptoms.

Some randomized controlled trials have shown a clear superiority of omega-3 administration over placebo (olive oil) in the reduction in depressive symptoms [117], while a Cochrane Review [118] did not reach a definitive conclusion because, although the statistical superiority of omega-3 is evident over placebo, the clinical significance of these results is still unclear. An update of this investigation [118], which evaluated over 30 studies of 1800 patients with depression treated with omega-3 or placebo, confirmed the superiority of omega-3 over placebo, although the difference, while statistically significant, is modest.

A subsequent meta-analysis confirmed the superiority over placebo of the eicosapentaenoic (EPA) and docosahexaenoic (DHA) mixture with >60% EPA at a dosage of 1–2 g per day [119].

We therefore agree with the Statement of the International Society for Nutritional Psychiatry Research (ISNPR), which, at a scientific meeting that brought together high-level experts convened specifically to reach a conclusion on the use of these substances in the treatment of depression, concluded that oral omega-3 supplementation may be recommended as an additional treatment in adults diagnosed with major depression [120]. In conclusion, there is a consensus on the use of omega-3 that can ameliorate depressive symptoms [121].

##### Zinc

This trace element is a necessary component for the synthesis of hundreds of enzymes and other proteins. It is required for the proper structure and maintenance of cell membranes and is involved in the regulation of the immune-neuroendocrine network. Its deficiency generates various consequences, including neurological problems. Zinc deficiency affects approximately 17% of the global population [122]. In a Japanese study, approximately 20% of the elderly and more than one-third of children under the age of 4 years reported zinc deficiency [123]. Elderly patients suffering from depression show zinc deficiencies in their blood. Studies in animal models and, in the last decade, studies in human subjects have documented the effectiveness of zinc supplementation in combination with standard drug treatment [124]. Zinc modulates the glutamatergic circuit by functioning as an N-Methyl-d-Aspartate (NMDA) receptor antagonist and enhancer of α-amino-3-hydroxy-5-methyl-4-isoxazolepropionic acid (AMPA) receptor activity. Its deficiency leads to hyperactivation of the NMDA receptor with increased inflammation and excitotoxicity with depressive effects [125,126]. However, brain zinc homeostasis is crucial because, while low levels are associated with neurological disorders such as Parkinson’s disease or multiple sclerosis, high levels have been found in Alzheimer’s disease [127]. Therefore, its possibly useful administration must be strictly controlled.

##### N-acetylcysteine

N-acetylcysteine has a powerful antioxidant effect as it is essential for the synthesis of glutathione, the major antioxidant at the cellular level. Glutathione (GSH in reduced form), in fact, plays a key role in the brain as an antioxidant. Recently, low GSH levels have been observed in several psychiatric diseases, including stress-related psychopathologies [128]. NAC has been used with positive effects in several psychiatric disorders, particularly in psychosis. With regard to depression, we have few and inconclusive studies, although we would like to point out that one study on patients with a major depressive episode, who had a chronic psychiatric disorder (on average, for almost 17 years) and therefore had a poor response to pharmacological treatment, concluded that supplementing standard treatment with NAC has favorable effects by reducing symptoms and improving patients’ functioning [129].

Remarkably, NAC has significantly higher anti-inflammatory efficacy than Aspirin and Minocycline, easily passes blood–brain barrier (BBB), and inhibits TNF, IL-1β, and IL-6 [130].

##### Vitamin D

A prospective study of 139 depression-free participants, of which 128 adults included in the UK Biobank, found a relationship between low serum vitamin D levels, measured at the start of the study (years 2006–2010), and the onset of depression in the follow-up period, carried out in 2016 [131]. In addition, associations with gestational and postpartum depression were studied. A systematic review of multicenter studies found that vitamin D deficiency is a risk factor predisposing to gestational and postpartum depression [132]. Confirmation came from research that analyzed 12 studies involving over 10,000 pregnant women and concluded that there is a relationship between low vitamin D levels and depression in pregnancy [133].

Although, in general, studies on the relationship between vitamin D and depression are not yet conclusive, we believe that clinicians should always keep an eye on the levels of this fundamental and multifaceted vitamin, which is a real hormone with documented anti-inflammatory and immune-modulating effects. When deficient, especially in pregnancy and postpartum, wisdom dictates that it should be promptly supplemented [134,135].

#### 3.2.4. Physical Activity

The scientific literature is unanimous in indicating positive effects of exercise on psychological symptoms and psychiatric disorders [136,137]. Therefore, several scientific societies, like the UK’s Royal College of Psychiatrists [138] and the European Psychiatric Association, have developed guidelines recommending “structured exercise training as an effective first-line treatment option for moderate depression and as an adjunctive intervention to improve symptomatic recovery in several mental illnesses” [139].

Two recent meta-reviews, which examined meta-analyses and systematic reviews on controlled studies, concluded that “there is converging evidence on the use of physical activity in primary prevention and clinical treatment in a spectrum of mental disorders” [96] including “depression, anxiety, attention deficit hyperactivity disorder (ADHD), substance use disorder and schizophrenia symptomatology” [140].

Finally, a meta-analysis that evaluated more than 200 studies with a total of more than 14,000 participants concluded that physical activity, in particular walking, running, and exercise has a level of efficacy which is overlapping to cognitive behavioral psychotherapy and superior to antidepressant pharmacology [141]. The same meta-analysis also found Yoga and Tai Chi to have comparable levels of effectiveness to drug therapy.

What are the mechanisms? Physical activity influences the brain both directly and via molecules released by muscle contraction. During physical activity there is a brain increase in serotonin, dopamine, endogenous opioids such as beta-endorphin, enkephalins, dynorphin [142]. There is growing interest in the role of serotonin in the hippocampus. A recent line of research hypothesizes that serotonin activates the 5-HT3 receptor, which is well present in the hippocampus and limbic system, which, in turn, activates IGF-1 with increased neurogenesis [143]. Recently, in an experimental model of Fibromyalgia, characterized by systemic inflammation with overexpression of NF-kB and high levels of IL-1β and TNF in the hippocampus, it was documented that physical activity reduces hippocampal inflammation and activates PGC-1α/FNDC5/BDNF lineage genes with positive effects on neurogenesis [144].

At the same time, with the contraction of skeletal muscle, a large flow of molecules reaches the brain, that some have called exerkines [145] including myokines, cytokines, chemokines, endocannabinoids, and other. Of particular interest is the role of the endocannabinoid anandamide, the concentration of which in the blood is greatly increased by physical activity [146]. Anandamide easily penetrates blood–brain barrier (BBB) and binds to the CB1 receptor, with antinociceptive and antidepressant effects [147]. Of interest is the role of irisin, an adipomyokine that acts via the PGC-1α/BDNF/IGF-1 line mentioned above with antidepressant and neurogenetic effects. The connection between muscle deficits (sarcopenia) and cognitive deficits in the elderly has been studied [148], influenced not only by myokines, but also by hormones released by the bone. Osteocalcin promotes monoamine synthesis, prevents hippocampal atrophy and slows cognitive decline in patients with Alzheimer’s disease. Osteopontin moderates neuroinflammation and intervenes in the clearance of amyloid-β (Aβ) [149].

#### 3.2.5. Pollution Indoor, and Outdoor

Pollution, climate, green spaces and mental health are related in multiple ways. Although extensive research work has still to be performed to define in precise terms the multiple relationships that have some evidence, the main facts may be, however, considered here and summarized.

This is mostly because chronic effects of pollution and global warming indeed have a marked and growing impact on mental health and therefore mental health professionals need knowledge and training to provide care in such enlarging population of patients.

Air pollution (measured in terms of several polluting chemical compounds, in particular NO2) and of solid pollutants like Particulate Matter (PM), is associated with an increased prevalence of depression [150]. These data are stronger in urban areas and in subjects of lower socioeconomic status.

The widespread presence of numerous pollution agents both outdoor (air, water, soil) and indoor (air, water supplies, chemicals used in house cleaning, etc.) without forgetting foods that are frequently highly processed and filled with chemicals used mostly to improve flavors, palatability and conservation. Toxicology and carcinogenicity data include a long list of chemicals that can be dangerous in many ways. Indoor, moreover, there is the frequent practice of tobacco smoke that is widely present and available to all the subjects living and breathing in the household, both to active smokers and to non-smokers receiving passively the same complex mixture of carcinogens and toxicants present in tobacco smoke.

On the contrary, the availability of green spaces and proximity of homes to green and natural areas is inversely associated with the prevalence of depression in the citizens having such a valuable proximity [150,151]. These effects are due to the higher opportunity to execute physical and recreational activities, having from the gardens and forests a significant compensation versus daily stress, having an easier promotion of social contacts and activities, thermoregulation and contrast to heat waves, cleaner air availability (because of plant oxygen production and reduction in CO2 removed by photosynthesis) and reduction in acoustic pollution: all elements available thanks to the proximity to green areas [151,152].

The World Health Organization (WHO) reported that in 2019, 1 in 8 people globally had a mental disorder, with anxiety (300 million) and depressive (280 million) disorders ranking as the most common disorders [153]. An already very solid and still increasing evidence indicates that exposure to air pollution increases the risk of anxiety and depressive disorders [154].

Climate change is showing direct and indirect effects on the prevalence of anxiety and depression in the population, by exacerbating environmental and socioeconomic determinants of mental health. Higher temperatures, heat waves, droughts, and fires reduce air quality, while simultaneously representing sources of distress and anxiety [153,155]. Therefore, the potential relationship between environmental exposures, social factors, and mental health could have broad population health impacts in a changing and ever more distressing global climate.

In the count of pollutants, it has recently emerged everywhere (in water, soil and atmosphere) that there is the presence of plastics that deteriorate and disperse as fragments: macroscopic (size from centimeters to millimeters), microplastics (size from 0.1 to 5 mm) and nanoplastics (size below 0.1 mm). Their biological effects range from modest to remarkable, when chemical compounds may interfere with endocrine functions and have carcinogenic potential. They appear to have also neurotoxic and behavioral effects, with possible depressive disorders and alteration of circadian rhythms [156].

This very recent experimental evidence demonstrated in zebrafish highlights that polystyrene microplastics (PS-MPs) are widespread pollutants in aquatic environments and that they may accumulate in various organs, including the brain, raising concerns about their neurotoxic effects, enacted mainly through neuroinflammation. Research by Binqui et al. [156] exposed zebrafish to environmentally relevant concentrations (25 and 250 μg/L) of PS-MPs for 40 days to investigate their impact on neurobehavior and underlying mechanisms. Results revealed that PS-MPs induced depression-like behaviors in zebrafish, characterized by reduced exploration, decreased locomotor activity, and altered social interaction. Histological analyses of brain tissue of exposed animals demonstrated PS-MP-induced neuropathological changes. Moreover, PS-MPs triggered neuroinflammation, evidenced by upregulated pro-inflammatory cytokines (IL-6, IL-1β), and disrupted the circadian rhythms, leading to altered expression of key clock genes. Furthermore, PS-MP exposure significantly altered neurotransmitter levels, decreasing dopamine, serotonin, norepinephrine, acetylcholine, tyrosine, and tryptophan. These findings provide solid evidence that PS-MPs induce depression-like behavior in zebrafish through mechanisms involving neuroinflammation, circadian rhythm disruption, and neurotransmitter imbalances, and stressing the potential ecological risks of PS-MPs and contributing to our understanding of the neurotoxicity of microplastics.

Another very recent experimental evidence links PS-MPs to anxiety and depression in mice [157]. In this study, experimental mice received by oral gavage micro plastics for 28 days and their behavior studied in comparison with control mice. PS-MPs exposed mice had a significant disruption of junctions between endothelial cells in the brain vessels and clear alterations in they behavior tests, including anxiety and depression-like changes with impaired social performance. Mitochondrial disfunction was also evident in exposed mice. These findings demonstrate clear neurotoxic effects of micro- and nano-plastics and raise the question of the highly possible involvement of human behavior perturbations with exposure to plastics environmental pollution.

Moreover, a new book chapter by Nava-Castro et al. [158] illustrates widely the relationship between pollution and mental disorders. We recommend such a reading for an extensive and updated discussion of this very important and very wide subject that here we have briefly summarized, concerning its major evidence.

## 4. Integrated Psychological and Biomedical Assessment and Treatment

Based on the pathophysiological framework outlined above, we propose to evaluate the human being as a whole, in its biological, psychological and social dimensions. An assessment was carried out by a team that includes internal medicine, psychology and psychiatry expertise, directing an integrated treatment centered on biological and psychological rebalancing, rationally using, for all disorders, a pool of pharmacological and non-pharmacological skills, such as psychotherapy and stress management practices, nutrition, nutraceuticals, physical activity and the promotion of sociality.

### 4.1. Assessment

It is well known that, since the third edition (1980) of The Diagnostic and Statistical Manual of Mental Disorders (DSM), diagnosis in psychiatry has been categorical, centered on the presence/absence of symptoms. The fifth edition (2013) maintained the symptomatic categorical approach with some openings in a dimensional sense. The debate was polarized between proponents of the ’spectrum’ and those of the ’category’. The National Institute of Mental Health (NIMH), noting the weakness of the DSM approach, proposed the initiation of a program to research new diagnostic criteria, which it called Research Domain Criteria (RDoC). The assumption of RDoC is that “unlike medicine, in psychiatry the diagnosis remains confined to subjective symptoms and observable signs” [159]. Hence, the need to equip psychiatry with objective diagnostic criteria as well. How? By classifying ’mental disorders as brain disorders’. This would seem to be nothing new. However, the NIMH leaders propose to move from the examination of symptoms to that of ’systems, social, behavioral, physiological, genetic’ and, on this basis, to stratify patients into categories organized on the integration of biological data and life experience. It is a proposal that undoubtedly highlights the weakness of the categorical approach of the current DSM, but which also appears somewhat confusing. Indeed, how is it possible to bring into the narrow category of ’disorders of the brain’, of the brain alone, all the data from a person’s social and individual life, as the researchers themselves propose?

A person who is frequently in a bad mood, who does not enjoy satisfying emotional and social relationships, who has little job satisfaction or who is unemployed, who has a high-calorie diet of low biological quality, who sleeps badly, does not exercise and has an inflammatory immune system, does he or she have a depressive disorder based on a brain disorder? Or is it not the person as a whole who has a disorder, which causes physical and mental suffering and limits his or her functioning?

It seems to us, therefore, that it is necessary to carry out an assessment, conducted jointly by a medical doctor and a psychologist, which examines the patient’s life history, highlighting psychological and biological critical passages.

The model, which is summarized in Table 1, is conceived as a basic guide for the assessment, which obviously does not make explicit more specialized aspects, which can easily be integrated. But, at the same time, it does not imply that the first visit is able to probe all aspects and with the indicated tools. Nor is it desirable for it to do so. The aim is to have from the outset a unified, albeit rough, view of the patient, which will be deepened over time to obtain a sufficiently profound knowledge of the patient, which will see the manifestations (symptoms) and the roots of suffering (life history).

The assessment probes three fundamental dimensions of the patient: the psychosocial one, with identifications of background characteristics and key passages in the course of life that may have marked the personality and the organism; the biomedical one which, with simple examinations and measurements, aims at investigating the stress system, the immune system and inflammation, metabolism, certain vitamins and minerals that are fundamental to said systems; the lifestyle one with regard to physical activity, nutrition, sleep, stress management, possible substance use and environmental pollution exposures over time. Having the three profiles together allows the therapeutic team to identify pathological connections and strengths to leverage in the therapeutic process.

### 4.2. Treatment

The main idea of integrated treatment is that changes on the biological level create the best conditions for action on the psychic system which, in turn, by reorganizing psychic constructs and behavior, positively influences biology, in a virtuous circle.

For example, it is known that in depression, and in other conditions of psychic suffering, physical and mental weakness and lack of energy are a very frequent trait. The asthenic condition, which sometimes makes it painful to carry out even the most trivial daily tasks, is undoubtedly associated with the depressed state, but it would be a mistake to think that it is a purely psychic phenomenon, a listlessness, which will therefore go away when the mood improves. In reality, energy deficiency has a physical basis that can depend on: 1. an excessive consumption of energy substrates by the immune system which, in depression, is overactive in an inflammatory sense; 2. by excessive energy consumption by the nerve cells that support a blocked and, at the same time, hyperactive brain: brooding, obsessive thoughts, a tiring dream activity; 3. damage to mitochondria [160,161], which are the energy source of cells, caused by inflammation and oxidative stress, also resulting from an unbalanced diet deficient in antioxidant substances, typical of the depressive state.

Therefore, helping the patient to improve his or her energy state, with a personalized medical prescription for nutrition and nutraceuticals or with other therapeutic tools with a low rate of adverse effects, can also be useful to improve self-perception and approach, with greater energy, the program of change proposed by psychotherapy.

In the previous paragraphs we have described the main scientific evidence that, in depression, supports the therapeutic role of diet, the use of natural substances (such as probiotics, vitamins, fish oil, minerals), and regular physical activity. Here, we would like to add an ancient neuromodulation technique: acupuncture, which also shows evidence of effectiveness in treating depression and related disorders. A systematic review and meta-analysis [162] that looked at 18 randomized controlled trials concluded that: 1. acupuncture treatment produced a significant improvement in sleep quality as measured by PSQI compared to standard medical treatment; 2. acupuncture combined with standard medical treatment has superior effects compared to medical treatment alone; 3. acupuncture combined with medical treatment has superior effects to medical treatment alone on depressive symptoms as measured by HAMD. Recently, a Randomized Controlled Trial in people with insomnia documented an improvement in symptoms associated with significant changes in functional connectivity between brain areas of the emotional network, as measured by fMRI resting-state functional connectivity (rsFC). Of note is the fact that the study was conducted with double control: healthy subjects and sham acupuncture versus real acupuncture [163].

A systematic review and meta-analysis [164], which examined 29 randomized controlled trials involving 2268 people diagnosed with major depression, concluded that: 1. acupuncture showed a significant clinical reduction in the severity of depression compared to both standard medical treatment and placebo acupuncture (sham); 2. the addition of acupuncture to standard treatment is superior to standard treatment alone; 3. a significant correlation was found between the number of acupuncture sessions and the reduction in the severity of depressive symptomatology, i.e., the more sessions (on average three per week), the better the results. Finally, adding acupuncture to drug treatment reducing the adverse effects of medication is relevant [165].

In conclusion, although there is a need for more high-quality research, acupuncture appears to be a safe and effective tool that can be available for the integrated treatment of depression [166].

## 5. A Clinical Case as an Example

GB is a 60-year-old woman who started the interview crying on the first visit, which was conducted jointly by FB (psychologist) and AGB (medical doctor). She suffered from Major Depression for more than 15 years and was under standard treatment: psychotherapy in combination with medication. However, the level of depressive symptomatology was high as we ascertained by administering two tests. She was severely overweight, had uncontrolled thyroiditis (elevated TSH) despite hormone therapy and uncontrolled osteoporosis (fractures) despite drug therapy with bisphosphonates and vitamin D; blood tests also showed a plasma cortisol value on the high end of the range, an inversion of neutrophil: lymphocytes ratio and a frank folic acid deficiency.

The story of GB: She was a wayward child, sad and angry with her mother, a cold woman. GB was quarrelsome with other children. She showed an unstable and aggressive character. A rebellious and quarrelsome adolescent, she was in boarding school from the age of 9 to 18. During this adolescent period, her first illnesses manifest as psoriasis and severe headaches. During her time studying at a university, she reported great well-being linked to her political commitments and the social climate of those years; during the preparation of her degree thesis, she experienced major panic disorder with agoraphobia and claustrophobia.

When she finished university and radically changed social climates and political commitments, she experienced sentimental difficulties, two voluntary abortions, and also gained weight following Hashimoto’s thyroiditis, which she tried to treat with homeopathic therapies and then with drugs. In post-menopause, she experienced arthritic degeneration (in the hands bilaterally) treated with cortisone therapy for short periods, and osteoporosis that was not controlled despite medication: vertebral and then costal fractures. Relationship difficulties persist, both sentimental and work related. Depressive disorder manifests itself clearly. Our diagnostic reasoning was based on the joint examination of the main aspects of the subject’s psychological and biomedical history, identifying the connections that may explain treatment-resistant depression (see Box 1).

Box 1. **Diagnostic reasoning.**

GB experienced an insecure aggressive attachment style as a child, which may explain the inflammatory-based disorders she experienced in adolescence: psoriasis and headaches [167,168].

Insecurity and aggression were mitigated in a supportive context where anger turned into political action. Entry into the world of work coincided with a phase of political reflux, where relational, sentimental and work-related difficulties manifested themselves, and other autoimmune pathologies (Hashimoto’s thyroiditis with hypothyroidism) and metabolic alterations (weight gain) arose.

Systemic inflammation and chronic stress, documented by high plasma cortisol levels, explain both the onset of clinical depression with associated sleep disorders, asthenia, anhedonia, sexual withdrawal, and osteoporosis and alterations in the leucocyte formula.

Therapeutic treatment was multi-layered: nutrition, nutraceuticals, acupuncture, anti-stress and meditative techniques, support for physical activity and psychotherapy. A low-calorie Mediterranean diet with a low glycemic index was prescribed. A galenic preparation containing vitamins and antioxidants was prescribed based on the blood tests performed and individual needs.

Myo-inositol 2 g/day was also prescribed to improve insulin sensitivity and for anti-oxidative purposes, bearing in mind the experimental applications for anxiety and depression. Probiotics in capsules (psychobiotics) were also prescribed to promote intestinal eubiosis and improve the function of the gut–brain axis, given the positive experimental findings on the use of probiotics in depression.

Melatonin in liquid form for evening sublingual intake (3 mg per night) was also prescribed to counter insomnia, promote falling asleep and restore a healthy sleep–wake rhythm. Melatonin, in addition to being an effective inducer of falling asleep, has also been shown to have an antidepressant effect probably linked to its antioxidant and central anti-inflammatory action [169].

GB also had a cycle of 14 acupuncture sessions in total, 30–45 min per session, three times a week. The protocol of the points where to insert the needles was determined on the basis of a Traditional Chinese Medicine diagnosis (anamnesis, pulse and tongue analysis).

**Figure 4:**
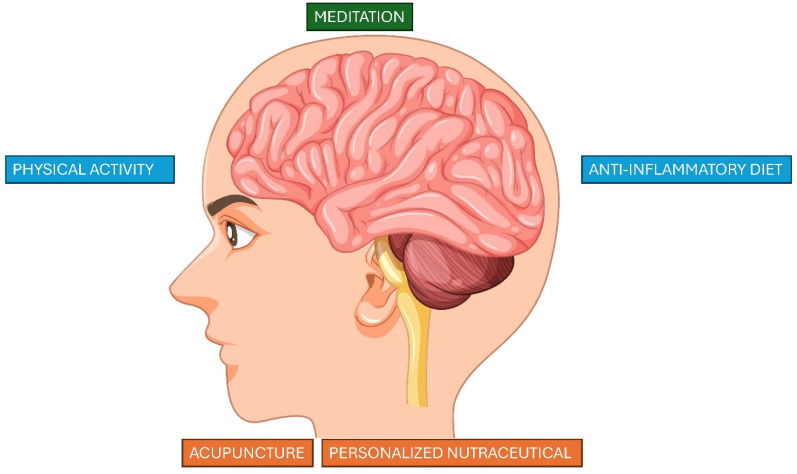
The integrated therapy followed by patient GB. Note that GB preferred to discontinue psychotherapy, which she had been following for years and with different psychotherapists, in favor of learning meditative practice according to the PNEIMED method. Obviously, this was GB’s preference, and others find themselves very well with psychotherapy and benefit from it.

GB followed a meditation course according to the PneiMed Method. A 30 h theoretical and practical course, designed by Antonia Carosella and Francesco Bottaccioli, which has been tested in controlled experimental studies [170,171] to significantly reduce symptoms of stress, anxiety, depression, somatization and inadequacy, and the level of perceived stress, both from a psychological and biological point of view, with a reduction in cortisol levels. The reduction in cortisol concentration is a relevant biological phenomenon since the main stress hormone has effects on the regulation of the immune system, metabolism and cortico–limbic circuits that manage emotions and mood [172]. Figure 4 illustrates the integrated therapy followed by patient GB.

Results after two and eight months later

Already in the first two months we saw a substantial improvement in mood: the tests document that the level of depression went from severe to moderate and the grade of severity from severe to moderate. GB started to lose weight, felt that she had more energy, was sleeping better, no longer experienced nocturnal hunger attacks, and had less joint and lower back pain. Eight months later, GB, despite the lock down imposed by the pandemic, continued to march towards wellness.

GB said she lived through the lockdown quite well, she worked from home, and that there were no shortage of moments of discouragement from living in complete solitude. She resumed work at the office and relations with colleagues improved. She received recognition for her professional skills. She felt more open with people and no longer felt the need to isolate herself from others. She was in a romantic relationship for approximately a month, after 10 years of loneliness, and the old trauma resurfaced, and she is still unable to have penetrative sex. Insomnia reappeared, which she kept at bay with hypnotics and melatonin. The weight loss continued (−11.5 kg since the beginning of the treatment, −6 cm waist circumference). Her lower back pain is sporadic, she does daily postural gymnastics and stretching, walks to work every day and takes long walks in the afternoon. She no longer has headaches, and meditates almost every day. She no longer has angry outbursts and reports that she no longer feels the sense of continuous internal tension.

Biological tests also show signs of the process of escaping from the malaise. Cortisol decreased from 19.1 to 16.1 μg/dL (normal range 4–19.5). The neutrophil:lymphocyte ratio, which was inverse (neutrophils 36%, lymphocytes 49%), resumed a march towards normality (neutrophils 46%, lymphocytes 41%). TSH, which was out of control (7.2 mU/L, normal range 0.15–3.5), normalized to 4.1 m/UL, and thyroid antibodies as well as insulin were significantly lowered. For details of start and end biological samples, see Table 2.

In conclusion, GB was studied and treated in an integrated way for severe major depression, and at follow-up at 8 months, she showed psychological rebalancing, new behavior, and a strong reduction in physical symptoms (see Table 3).

## 6. Discussion 

We must acknowledge that the model on which psychiatry has been based for the last five decades is exhausted. From the early 1980s until recent years, biological psychiatry was a scientific ideal that conquered psychiatrists, doctors, and the public. The basic concept is that mental disorder is a brain disorder, depression is the result of serotonin deficiency, schizophrenia is the result of dopamine alteration, etc. The treatment is therefore medical, meaning pharmacological treatment. Just as diabetes, which is the product of an insulin deficiency, is treated by administering insulin, so depression, which is the product of a serotonin deficiency, is treated by administering a drug that enhances its brain availability—this is the leitmotif. One of the consequences expected is that the biological revolution will sweep away the old talk therapy.

Things went very differently. Psychopharmacological research has not produced substantial innovations, nor have there been convincing biological explanations of mental states in health and disease. The king is naked, biological psychiatry has failed. Caleb Gardner and Arthur Kleinman write that "Biologic psychiatry has thus far failed to produce a comprehensive theoretical model of any major psychiatric disorder, any tests that can be used in a clinic to diagnose clearly defined major psychiatric disorders, or any guiding principle for somatic treatments to replace the empirical use of medications." [173]—an all-round failure: in the etiopathogenesis, in the diagnosis, in the therapy.

A first attempt to come out of the crisis was based on the re-evaluation of psychotherapy, which undoubtedly has a higher effectiveness in the treatment of depression than drugs in the medium to long term. But even this approach, while useful in reducing relapse after partial remission of depression, is not able to significantly improve quality of life in the long run [174]. Another ongoing attempt is to pharmacologically address the inflammation that, in a substantial number of cases, accompanies depression [175]. However, the experiment did not give satisfactory results [176], although attempts are still in progress [177]. The last pathway, which is also gathering enthusiasm among psychotherapists, involves psychedelic drugs: psilocybin, lysergic acid diethylamide, and 3,4-methylenedioxymethamphetamine. At the moment, only the first has been authorized by the FDA. However, its antidepressant efficacy in controlled trials is modest and is only recorded with high doses; moreover, it must be considered that the use of psilocybin is carried out in a psychotherapy-assisted context, which could therefore alter the degree of effectiveness, enhanced by psychotherapy [178]. In conclusion, the different ways out of the crisis of the biological psychiatry do not seem to be able to solve it. They may increase the pharmacological armamentarium of the psychiatrist, but we are far below the needs posed by an enhancement of the effectiveness of care, in a context of increasing depressive disorders, particularly among young people.

A new pathophysiological model that attempts to understand the complexity of human beings and mental disorders is urgently needed. The proposal we put forward in this paper is based on the paradigm of psychoneuroendocrineimmunology, which sees the human being as a network of complex systems, the psychic, the nervous, the endocrine, the immune, the metabolic in a relationship of mutual influence [44,179,180].

The PNEI paradigm explains why mental and mood status can be influenced by the activity of the immune system, as well as of digestive and metabolic system, gut microbiota, musculoskeletal and the stress system. The brain is very receptive for signaling from the body. A mouse cortical neuron expresses RNA transcripts for more than 60 receptors for neurotransmitters, metabolites, hormones, peptides, cytokines and other substances produced by the body [181]. Psychic state, therefore, emerges from the complex network of human networks, which are different from physical networks. Signal coding in the brain, for example, is implemented by a pattern of multiple signals coming from different networks, unlike physical networks (Internet, for example). Moreover, unlike physical networks, in biological networks there is no separation between function and structure [182]. In biological networks, function can change structure.

The function is connected to the psychic system. Regarding depression, we have evidence of pathological change in important brain circuits, such as salience networks, connected to attention but also to the rumination typical of the depressed person or who will develop depression [22]. In contrast, psychological interventions, such as cognitive psychotherapy and mindfulness, as well as lifestyle interventions have an effect on reducing inflammatory cytokines [183]. In our study, we also reviewed scientific evidence on the role of nutrition, physical activity and personalized nutraceuticals in reducing depressive symptoms. Therefore, we believe that it is necessary to trace a path to overcome the crisis of psychiatry by adopting a systemic approach, combining medicine and psychology.

Personalized diagnosis and treatment of depression should identify in the patient’s personal history the sources of the altered networks, some of which may be remote, others current. The scientific literature, presented in this paper, documents that adversity in the early stages of life can have long-lasting dysregulating effects on psychic and biological networks from which neuroinflammation originates. Thus, distress and toxic stress, intrapsychic conflicts throughout life, inflammatory eating, sedentary lifestyle, and exposure to environmental pollution epigenetically mark nerve and immune cells and favor the emergence of depressive disorder which, in turn, will aggravate the imbalance of the psychophysical network. 

We note that the application of systemic psychology and medicine to the study of the individual complexity can be greatly facilitated by the application of artificial intelligence (AI) that could enhance multi-omics research, a new biological profiling approach, that could provide a picture of the molecular processes occurring in stress [184], in nutrition [185], and in physical activity [186] identifying unexpected connections that can play an important role in the patient.

In the clinical case of severe depression we presented, without the aid of AI, we found that the inducer of the process of change towards healing was neither drug nor psychotherapy, but a mix of biological and psychological actions. The biological actions, obtained by various tools (nutrition, precision nutraceuticals, acupuncture), provided energy to the patient and lowered inflammation by regulating the immune system. So, a new interoceptive state was realized. On this new perception of self, learning meditative and stress management practices facilitated the implementation of self-management of emotions and the practice of new health behavior.

Medicine and psychology are thus two ways of knowing and modulating the human organism in its entirety. Their scientific and clinical collaboration and cross-fertilization are realistic and necessary, and our opinion is the only way to make a leap forward in the prevention and treatment of clinical depression [187,188].

There is no shortage of obstacles concerning: 1. the scarcity of funding for PNEI clinical research, which the industry, mistakenly in our opinion, does not consider profitable for pharmacological research; 2. the backwardness of the experimentation of integrated treatment models; 3. the resistance of political, educational and professional institutions to accept radical change. However, this study offers a fresh, integrative perspective on depression by moving beyond traditional neurotransmitter theories. It combines biological, psychological, and social factors, providing a more comprehensive understanding of the disorder. The approach is innovative, grounded in psychoneuroendocrineimmunology, and aims to bridge critical gaps in current treatment strategies. By emphasizing an interdisciplinary framework, this study has the potential to enhance both theoretical knowledge and clinical practice, ultimately improving patient outcomes (from the Academic editor suggestions, whom we thank).

## 7. Conclusions

The reductionist model in psychiatry is unable to explain the complex origin of mental disorders and does not provide clinicians with adequate diagnostic and therapeutic tools to treat mental suffering [173]. The reductionist model has stagnated pharmacological research, which has not produced any substantial innovation for approximately half a century, particularly for the treatment of depression, the level of effectiveness of which is modest [17] and antidepressant drug mechanisms shrouded in fog [10]. It has become common sense among researchers and clinicians to move from a ’one-size-fits-all’ therapy to a personalized one. However, if the paradigm of reference is not changed, the desired precision psychiatry risks being reduced to the choice of the mix of psychiatric drugs to propose to the patient, to which traditional or new-generation anti-inflammatory drugs should be added. The risk that precision psychiatry runs, in our opinion, is to remain in the reductionist trap of searching for the molecular switch that resets the whole system and switches off the disease—a trap from which it is necessary to exit definitively.

This study shows that a new pathophysiological model is scientifically mature, as basis for a new type of treatment of depression and, probably, of other psychiatric disorders, which replaces the old reductionist model and the standard therapy, even of a “combined type” (pharmacotherapy and psychotherapy together), which is of little efficacy. 

The sources of depression are multiple and personal, which must be recorded in detail in the joint medical and psychological assessment. The leap forward in the prevention and therapy of depression consists of proposing models of personalized integrated treatment that treat the different factors of the disorder, biological, psychological, and behavioral, as they occur in the personal mix of the depressed subject.

The paradigm shift based on psychoneuroendocrineimmunology began a few decades ago. “Outcome will depend upon those who have the courage to try new paths and the wisdom to provide the necessary support” [30], p. 135.

## Figures and Tables

**Table 1:** Basic Assessment by Psychological and Medical Doctors together.

Psychological	Biomedical	Lifestyle
Loneliness Social isolation Interpersonal conflict Violence, abuse, neglect Adverse childhood experiences Lifetime stressor exposure TEST PHQ-9, GAD-7 in absence of MDD diagnosis PROMIS Depression Short Form-8 to confirm and score MDD	Biological markers Saliva: Cortisol 2 measures; awakening and 10 p.m. Cytokines proinflammatory (IL-1β, IL-6, TNF-α) Blood: complete blood count, insulin, glucose, glycated hemoglobin, CRP, thyroid hormones (TSH included), liver functionality, Vit. D3, Vit. B12, folic, magnesium Anthropometric and physiological measures BMI, WHR, HR, HRV, BP	Physical activity (IPAQ questionnaire) Nutrition (Mediterranean diet, PREDIMED Questionnaire) Sleep (Pittsburgh sleep quality index) Pollution (indoor, outdoor)

BMI, Body Mass Index; BP, Blood Pressure; CRP, C-reactive Protein; HR, Heart Rate; HRV, Heart Rate Variability; MDD, major depressive disorder; WHR, Waist–Hip Ratio.

**Table 2:** Extract of the most significant results of WBC tests performed on GB at the beginning and after 8 months.

Start	End
Cortisol 19.1 μg/dL (n.r. 4–19.5) 8.00 a.m.	16.1 μg/dL
Neutrophils 36%	46%
Lymphocytes 49%	41%
TSH 7.2 mU/L (n.r. 0.15–3.5)	4.1 mU/L
fT3 2.7 pmoli/L (n.r. 3–8)	3.1 pmoli/L
Ab anti-TG 1029 UI/mL (n.r. < 116)	221
Triglycerides 160 mg/dL (n.r. 50–150)	110 mg/dL
Folic acid 3.7 ng/mL (n.r. 3.9–26.8)	10 ng/mL
B12 vitamin 201 pg/mL (n.r. 150–900)	337 pg/mL
Insulin 14 μUI/mL (n.r. < 25)	9 μUI/mL
Glycemia 98 mg/dL (n.r. < 100)	84 mg/dL

n.r.: Normal range.

**Table 3:** Tailored PNEI-based therapy led GB towards recovery.

Psychological Rebalancing	New Behavior	In
1. Overcame the lockdown 2. Improved relations with colleagues 3. New love story 4. Has no outbursts of anger 5. No longer feels internal tension	1. Meditates regularly 2. Daily exercise 3. Long walks 4. Walks to work	1. No more headaches 2. Sporadic lower back pain 3. Sustained weight loss

## Data Availability

Data are contained within the article and Supplementary Materials.

## Supplementary Materials

The following supporting information can be downloaded at: https://www.mdpi.com/article/10.3390/ijms26062759/s1.
