# Supplementary material for: Rethinking Depression—Beyond Neurotransmitters: An Integrated Psychoneuroendocrineimmunology Framework for Depression’s Pathophysiology and Tailored Treatment
Source: Int J Mol Sci. 2025 Mar 19;26(6):2759. doi: 10.3390/ijms26062759 (PMC11943243; doi:10.3390/ijms26062759)
Supplement: Supplementary file 1 [file ijms-26-02759-s001.zip › ijms-3490232-supplementary.pdf]

Supplementary.

Rise and decline of depression standard vision: brief history

It is known that the discovery of drugs active on the brain was accidental (serendipity). It is due to the insatiable mind of Henri Laborit (1914-1995), a surgeon who hypothesized that many complications during operations resulted from the excessive release of histamine; He then asked a pharmaceutical company to synthesize an antihistamine to be used in an intraoperative session. He experimented with promethazine and chlorpromazine. The latter had an evident anti-anxiety effect on patients. He proposed to two psychiatric colleagues to experiment with the substance, which gave good results in the control of manic symptoms and in the so-called positive symptoms of schizophrenia. In 1954, chlorpromazine was put on the market. In those same years, a similar compound, imipramine, did not prove effective as a "neuroleptic" but as an antidepressant: this was the starting point for the generation of so-called tricyclic antidepressants.

One of the many consequences of the increasingly extensive use of psychotropic drugs, in our opinion the most relevant, was that theories were built on the functioning of the brain that could explain the effects of the new molecules, to which, at the end of the seventies, selective serotonin and then norepinephrine reuptake inhibitors were added. Tricyclic antidepressants and SSRIs (fluoxetine, sertraline, paroxetine) and SNRIs (venlafaxine and others) work – according to these theories – because they prevent the presynaptic reabsorption of neurotransmitters such as norepinephrine, serotonin, dopamine; some have anticholinergic effects, others inhibit enzymes, others modulate the serotonin receptor family. Thus, the theory that has dominated for the past four decades considers depression to be the result of a chemical imbalance, particularly of neurotransmitters such as serotonin and norepinephrine. It is the so-called monoaminergic theory of depression that, with more difficulty, has also been tried to be applied to psychoses (dopamine receptor alterations). In the early eighties, biological psychiatry became a scientific ideal in the United States that quickly conquered psychiatrists, doctors, and the public.

The historian of science Anne Harrington recalls that 1984 was a turning point [13]. The influential psychiatrist Nancy Andreasen publishes a book that is the manifesto of biological psychiatry, The Broken Brain: The biological Revolution in Psychiatry, where one can read: "psychiatry is undergoing a revolutionary change, of realignment with the biological traditions of medicine" [13] (p. XII). Alongside psychiatrists are opinion leaders, science journalists, such as Pulitzer Prize winner John Franklin who declares: "The future of psychiatry does not rest on the old speech therapists and analysts, but on a new generation of clinical scientists who work quietly in the laboratory, dissecting the brains of mice and humans, playing with chemical formulas that reveal the secrets of the mind" (ibid.).

Biological psychiatry is based on the following concepts: 1) mental disorders are disorders caused by a malfunction of the brain; 2) the causes are genetic; 3) the only therapy is pharmacological; psychotherapy can only be supportive. In the nineties and throughout the first decade of the twenty-first century, scientific publications multiplied with the aim of documenting the solidity of the pillars of the paradigm of biological psychiatry, as well as textbooks, for use not only by psychiatrists but also by psychologists who put the following concepts at the basis of their teaching: "The fundamental idea that psychiatric problems arise from psychological causes is definitely abandoned. The genesis of psychiatric pathologies is now discussed in organic terms" [14] (p. 8); genetics is the key to understanding psychiatric disorders, but also behavioral problems because "genetics plays the main role in behavior" [15].

With the beginning of the new century, evidence has accumulated that does not confirm the three pillars of the standard view.
